# Efficiency of IRAP and ITS-RFLP marker systems in accessing genetic variation of *Pyrenophora graminea*

**DOI:** 10.1590/S1415-47572010005000041

**Published:** 2010-06-01

**Authors:** Imad Zein, Mohammed Jawhar, Mohammed Imad Eddin Arabi

**Affiliations:** Department of Molecular Biology and Microbiology, AECS, DamascusSyria

**Keywords:** *Pyrenophora graminea*, IRAP, internal transcribed spacer, rDNA

## Abstract

The usefulness of IRAP (inter-retrotransposon amplified polymorphism) and ITS-RFLP (restriction of PCR-amplified internal transcribed spacers of the rDNA) markers in the analysis of 39 *Pyrenophora graminea* isolates was determined. Each marker system could discriminate between all of the isolates in detecting polymorphism, albeit with variable efficiency. IRAP and ITS-RFLP produced 85% and 77% polymorphic bands, respectively, with a corresponding mean polymorphic information content (PIC) of 0.38 and 0.36. The IRAP marker index ratio (2.41) was higher than ITS-RFLP (1.50). On one hand, the quality nature of data (QND) was higher for ITS-RFLP (0.169) than IRAP (0.093). However, correlation between both marker similarity matrices was significant (*r* = 0.34, p < 0.05). These findings suggest their combined use in phylogenetic analysis. To our knowledge, this is the first report of a comparison involving these two advanced DNA marker systems.

*Pyrenophora graminea* [anamorph *Drechslera graminea* (Rabenh. *ex.* Schltdl.) Ito], the seed-borne pathogen responsible for leaf-stripe in barley (*Hordeum vulgare* L.), inflicts heavy losses on crops (Porta-Puglia *et al.*, 1986; Arabi *et al.*, 2004). Several studies on morphological, physiological and biochemical aspects have already been undertaken (Zriba and Harrabi, 1995; Jawhar *et al.*, 2000).

Traditionally, the classification of *P. graminea* isolates, besides requiring a certain expertise in taxonomy, may be further complicated by the inherent variation in morphological features among isolates, besides being time consuming, especially in those cases where similar species may be present in one and the same field (Gatti *et al.*, 1992).

Over the years, the methods for detecting and assessing genetic diversity have extended from the analysis of discrete morphological traits to those of biochemical and molecular origin. Two classes of molecular markers which have received much attention in recent studies on genetic diversity in natural populations, are inter-retrotransposon amplified polymorphism (IRAP) (Kalendar *et al.*, 1999; Pasquali *et al.*, 2007), and restriction fragment length polymorphisms (RFLP) of PCR amplified internal transcribed spacer (ITS) regions (ITS-RFLP) (Hsiang and Wu 2000; Nilsson *et al.*, 2008). The usefulness of these two markers types extends to resolve genetic variation among species within a genus or among populations (Redecker *et al.*, 1997; Martin and Rygiewicz 2005; Branco *et al.*, 2007).

Despite the general interest, it is not clear whether these two markers have comparable power for quantifying differentiation among populations. Thus, it would be of interest to determine whether IRAP and ITS-RFLP markers are equally efficient at detecting genetic patterns existent among *P. graminea* isolates. However, differences would suggest that one marker may be more appreciated for detecting isolation, which has implications for the use of either type of marker for defining demographically independent management units (Mortiz, 1994).

The present study aimed to evaluate the usefulness of both markers in assessing and analyzing the nature and extent of genetic diversity among isolates of *P. graminea* collected from various regions in Syria.

The 39 monosporic isolates of *P. graminea* used in the study were identified, cultivated, and maintained as described by Arabi *et al.* (2002, 2004). They were isolated from leaf-stripe infected barley leaves, originating from various regions in Syria, and selected from among 93 isolates, according to morphological and physiological criteria (virulence). The isolates were grown separately in 9 cm Petri dishes containing potato dextrose agar (PDA, DIFCO, Detroit, MI. USA), and incubated for 10 days at 21 ± 1 °C in the dark to facilitate mycelia growth.

Genomic DNA was extracted from fungal cultures as previously described (Arabi and Jawhar, 2007).

ITS regions and 5.8S rDNAs were amplified for all the isolates using the ITS1 (5' TCCGTAGGTGAACCTGCGG 3') and ITS4 (5'TCCTCCGCTTATTGATATGC 3') primers designed by White *et al.* (1990). The amplification protocol was as described by Arabi and Jawhar (2007). In separate reactions, 10 μL of PCR reaction were digested for 3 h with six different endonucleases (*Alu*I, *EcoR*1, *Bsur*I, *Bam*HI, *Rsa*I and *Hind*III), according to manufacturer's recommendations (MBI Fermentas, York, UK). DNA fragments were size-fractionated by electrophoresis through 1.5% agarose gels. The sizes were determined by comparison with their molecular weight relative to a DNA ladder (Q.BIOgene).

The IRAP method was used for retrotransposon amplification, as described by Kalendar *et al.* (1999). Primer sequences, as well as retrotransposon type and orientation are shown in Table 1. PCR was carried out using the method described by Jawhar and Arabi (2009). Amplified products were electrophoresed in a 2% agarose gel using a 1 x Tris-borate-EDTA buffer (100 mM Tris-HCl/L, pH 8.3, 83 boric acid/L, 1 mM EDTA/L) at 100 V. Subsequently, the gels were stained with ethidium bromide solution and visualized under ultraviolet illumination. The sizes of the amplified products were determined as mentioned above.

ITS-RFLP and IRAP banding profiles were scored for the presence (1) or absence (0) of bands. The experiments were repeated twice for each isolate and both markers, so as to confirm repeatability and remove monomorphic bands from the analysis. The data were converted to a Jaccards similarity (Jaccard, 1908) coefficient, which was used to construct a dendrogram by the unweighted pair-group method with arithmetic averages (UPGMA) utilising the software package Phylip 3.7 (Felsenstein, 1985). The polymorphism information content (PIC) was calculated for each locus according to Anderson *et al.* (1993), which provides an estimate of the discriminating power of a locus by taking into account the number of alleles generated by each reaction unit and their frequency distribution in the population. The percent of polymorphic markers (β) was estimated by dividing the number of polymorphic markers by the number of obtained markers. The multiplex ratio (MR) is defined as the number of bands per reaction unit, and the effective multiplex ratio EMR as the product of MR with the fraction of polymorphic markers. Marker utility (MI) for genetic studies was estimated as a marker index according to Powell *et al.*, (1996) and Milbourne *et al.* (1997).

As PIC values are equal to gene diversity in binary marker systems, the effective number of alleles per marker was calculated as the respective reciprocal of the PIC value. The Mantel test (Mantel, 1967) was applied to ascertain the significance of correlations between pairwise genetic similarities in both marker systems. The probability of calculated correlation was estimated based on 1000 random permutations. These computations were carried out using the Arlequin software package (Excoffier *et al.*, 2005). The quality nature of data (QND) of the marker system and the effective marker index EMI as an overall criterion for the utility of molecular markers were calculated according to Varshney *et al.* (2007).

Selected IRAP bands were cut with a surgical blade and purified with a QIAgene gel extraction kit according to manufacturers recommendations. Sequencing was carried out on a Genetic Analyzer (ABI 310, Perkin-Elmer, Applied Biosystems, USA). Each sequence was identified by homology search using the Basic Local Alignment Search Tool (BLAST) program (Altschul *et al.* 1997) against the GenBank nonredundant public sequence database.

PCR amplification with the specific primers ITS1 and ITS4 yielded single DNA fragments present in all isolates with ~ 650 bp in size, which is in agreement with the results obtained by a previous study (Arabi and Jawhar, 2007). Fingerprints generated from the five restriction digestions of the nrDNA ITS region denoted high levels of intraspecific variation within the *P. graminea* population. A total of 354 scorable DNA bands were scored, 274 of which (77%) being polymorphic, while the number of bands in isolates varied from 3 to 5.

Based on IRAP patterns, 534 bands were obtained, 454 (85%) of which were polymorphic, whereas the number of DNA bands in isolates varied between 4 and 15 (Figure 1). This is sustained by the findings of Taylor *et al.* (2004), who found a presence of high copy numbers of *Pyggy*-like sequences in the *P. graminea* genome by using a primer derived from the LTR (long terminal repeat) of the *Pyggy* retrotransposon isolated from this fungus. However, the sequence of one IRAP fragment, when using a Sukkula primer, showed similarity 19/27 (70.4%) to the LTR of the *Pyggy* retrotransposon (AF533703.1). Similarity began from position 34, as position 4 was a G instead of an A, and position 6 an A instead of a G. Furthermore, bases were missing at positions 13 and 24.

In addition, the *P.graminea* LTR sequence and a rice cDNA clone (Accession No. AK058381) were significantly similar, as attested by 117/147 bp identity (80%). Homology was also evident between *P.graminea* LTR and an *Alternaria alternata* LTR (AB025309), with 1556/2096 bp identity (74%). These results indicated the capability of LTR-specific primer to amplify in different target species.

On the other hand, both markers were highly repeatable, although QND was 0.191 for ITS-RFLP markers and only 0.141 for IRAP. PIC values were 0.376 and 0.355 for IRAP and ITS-RFLP, respectively. Furthermore, IRAP markers generated a substantially higher number of markers (7.80) and a superior marker index (2.41) than ITS-RFLP (Table 2).

The UPGMA dendrogram generated from IRAP and ITS-RFLP data demonstrated that isolates clustered into five groups for both markers by a similarity index of 0.341 for IRAP and 0.460 for ITS-RFLP (Figure 2). The correlation between IRAP and ITS-RFLP similarity matrices was moderate but significant (*r* = 0.34, p < 0.05).

The usefulness of a molecular marker technique depends upon both the polymorphic information content (PIC) of the markers and the number of markers generated by each primer (Varshney *et al.*, 2007). Even though both IRAP and ITS-RFLP markers exhibited comparable PIC values, owing to the higher number of markers per assay, the MI of IRAP was 2.41 higher than for ITS-RFLP (Table 2). Bernardes *et al.* (2007) compared the performance of REMAP, a retrotransposon based maker technique, and ISSR with the fungus *Magnaporthe grisea* and has found an MI of 1.54 and 4.25, respectively. These marker systems are similar to IRAP and ITS-RFLP, in that they are PCR-based, anonymous and dominantly inherited. They also depend on repeated patterns in the genome to provide annealing sites for universal primers.

The results showed that band quality could have benefited from the additional restriction step following amplification in the ITS-RFLP protocol, thereby leading to a clearly defined band pattern. The documentation capabilities of band information produced by both marker assays in gene bank systems were comparable, as they were equally evaluated. This was placed on par with the widely used AFLP markers (Varshney *et al.*, 2007), which are far inferior to single locus markers such as SSRs or SNPs with unique primers for each locus. On considering both quantitative and qualitative attributes, IRAP turned out to be superior to ITS-RFLP, as depicted by a more effective marker index (0.338 and 0.255, respectively).

Researchers have examined the existence of correlations between various molecular marker techniques in *Fusarium oxysporum* f. sp. *lentis*. Belabid *et al.* (2004) reported similar genetic relationships through RAPD and AFLP analysis. In barley, Russell *et al.* (1997) found that RFLP and AFLP, but not SSR, were correlated. In the present study, we found significant and moderate correlation between IRAP and ITS-RFLP in *P. graminea* pathogen according to the Mantel test, which confirmed in the partially conserved dendrogram topologies inferred from each of the similarity matrices (Figure 2). The moderate correspondence between these markers could possibly be attributed to different amplification targets in the *P. graminea* genome.

To our knowledge this is the first comparative report on the two advanced IRAP and ITS-RFLP genetic marker systems. The present study emphasized that, besides their effective employment, both of these DNA markers may furnish comparable results in assays of genetic differentiation among *P. graminea* isolates. Furthermore, due to the specific advantages of each marker, the combination of both marker systems can give us greater confidence that the delineated patterns are real, through drawing on results from multiple genetic systems (Allendorf and Seeb, 2000).

**Figure 1:**
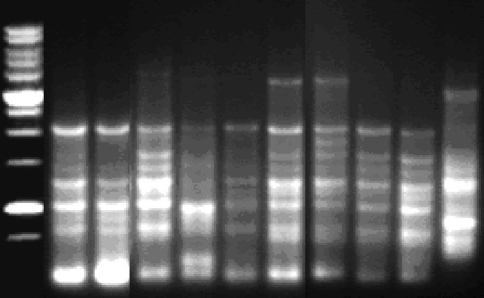
Agarose gel electrophoresis of IRAP (primers 3'LTR and 5'LRT1) in 10 *P. graminea* isolates. M – Marker ladder 1 kb.

**Figure 2:**
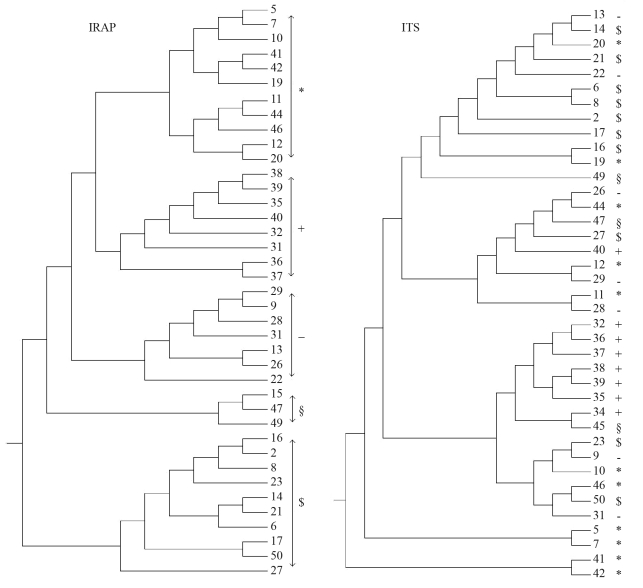
UPGMA dendrogram of 39 *P. graminea* isolates showing the agreement in clusters obtained by IRAP and ITS-RFLP markers. For methodology see text. (IRAP clusters were identified by the symbols (*, +, $ and §), and individuals in ITS clusters tagged with the corresponding symbol of their IRAP cluster).

## Figures and Tables

**Table 1:** Primer name, retrotransposon type, position and sequence.

Name and orientation	Retrotransposon type	Accession	Position	Sequence
3'LTR →	*BARE-1*	Z17327	2112-2138	TGTTTCCCATGCGACGTTCCCCAACA
LTR6149 →	*BARE-1*	Z17327	1993-2012	CTCGCTCGCCCACACATCAACCGCGTTTATT
LRT6150 ←	*BARE-1*		418-439	CTGGTTCGGCCCATGTCTATGTATCCACACATGTA
5'LRT1 ←	*BARE-1*	Z17327	1-26	TTGCCTCTAGGGCATATTTCCAACA
5'LRT2 ←	*BARE-1*	Z17327	314-338	ATCATTCCCTCTAGGGCATAATTC
Sukkula →	*Sukkula*	AY054376	4301-4326	GATAGGGTCGCATCTTGGGCGTGAC
		AY054373		
Nikita →	Nikita	AY078074	1-22	CGCATTTGTTCAAGCCTAAACC
		AY078075		

**Table 2:** Estimates of key statistics for evaluating the performance of IRAP and ITS-RFLP markers in 39 isolates of *P. graminea*.

Component	IRAP	ITS
Nr. of assay units	5 (primer comb.)	6 (enzymes)
Total nr. of bands	534	354
Polymorphic bands (percent)	454 (85%)	274% (0.774)
Percent polymorphic loci (β)	94%	0.89%
PIC^*^ (min; average; max)	0.139; 0.376; 0.500	0.289; 0.355; 0.500
Nr. of loci PIC > 0.3	27	14
Mean effective allele number	1.669 ± 0.357	1.639 ± 0.378
Multiplex Ratio (MR)	7.8	4.75
Effective Multiplex Ratio (EMR)	6.4	4.25
Marker index (MI)	2.41	1.50
Effective Marker Index (EMI)	0.338	0.255
Gen. simil. (min; average; max)	0.111; 0.341; 0.857	0.118; 0.460; 0.900
Quality nature of data (QND)	0.093	0.169

^A^Value considering only polymorphic markers.
